# Antigenic and immunogenic properties of recombinant proteins consisting of two immunodominant African swine fever virus proteins fused with bacterial lipoprotein OprI

**DOI:** 10.1186/s12985-022-01747-9

**Published:** 2022-01-21

**Authors:** Guanglei Zhang, Wei Liu, Zhan Gao, Yanyan Chang, Sicheng Yang, Qian Peng, Sudan Ge, Bijing Kang, Junjun Shao, Huiyun Chang

**Affiliations:** State Key Laboratory of Veterinary Etiological Biology, National Foot-and-Mouth Diseases Reference Laboratory, Lanzhou Veterinary Research Institute, Chinese Academy of Agricultural Sciences, Lanzhou, Gansu Province China

**Keywords:** African swine fever virus, Immunomodulation, Immune response, OprI, Recombinant fusion proteins, Vaccine

## Abstract

**Background:**

African swine fever (ASF) is a highly fatal swine disease, which threatens the global pig industry. There is no commercially available vaccine against ASF and effective subunit vaccines would represent a real breakthrough.

**Methods:**

In this study, we expressed and purified two recombinant fusion proteins, OPM (OprI-p30-modified p54) and OPMT (OprI-p30-modified p54-T cell epitope), which combine the bacterial lipoprotein OprI with ASF virus proteins p30 and p54. Purified recombinant p30 and modified p54 expressed alone or fused served as controls. The activation of dendritic cells (DCs) by these proteins was first assessed. Then, humoral and cellular immunity induced by the proteins were evaluated in mice.

**Results:**

Both OPM and OPMT activated DCs with elevated expression of relevant surface molecules and proinflammatory cytokines. Furthermore, OPMT elicited the highest levels of antigen-specific IgG responses, cytokines including interleukin-2, interferon-γ, and tumor necrosis factor-α, and proliferation of lymphocytes. Importantly, the sera from mice vaccinated with OPM or OPMT neutralized more than 86% of ASF virus in vitro.

**Conclusions:**

Our results suggest that OPMT has good immunostimulatory activities and immunogenicity in mice, and might be an appropriate candidate to elicit immune responses in swine. Our study provides valuable information on further development of a subunit vaccine against ASF.

## Background

African swine fever (ASF), which is one of the “notifiable diseases” listed by the World Organization for Animal Health, is a highly contagious hemorrhagic disease that causes serious economic losses worldwide [1, 2]. Because there is no effective vaccine, ASF causes devastating disease in domestic pigs and remains a major threat to the global pig industry [3]. ASF is caused by African swine fever virus (ASFV), a large enveloped DNA virus. ASFV belongs to the genus *Asfivirus* within the *Asfarviridae* family [4, 5]. Its double-stranded DNA genome has 170–193 kbp and contains more than 150 open reading frames [6]. Depending on the sequence of the *B646L* gene, which encodes capsid protein p72, ASFV is currently classified into 24 different genotypes, all of which have been detected in Africa [7]. The genotype II ASFV emerged in Georgia in 2007 and since then has spread from the Caucasus region to many countries of Eastern Europe [8]. In 2018, the virus was transmitted to pigs in China and other Asian countries [9, 10]. By the end of 2020, more than 180 outbreaks of ASF had occurred in 32 provinces of China (http://www.oie.int/). The disease is still prevalent in China and poses a serious threat to the large domestic pig population. Accordingly, a safe and effective vaccine against ASF is urgently needed.

Various strategies have been evaluated for the development of ASF vaccines [11, 12] but, unfortunately, all attempts to develop safe and effective vaccines against ASF have so far been unsuccessful [11, 13]. To date, vaccines containing inactivated viruses have failed to protect animals from ASF, even in the presence of modern adjuvants [14, 15]. Attenuated or low virulent ASFV strains could elicit protective immune responses to homologous, or even heterologous, virulent ASFV strains in swine [16, 17], offering in some cases the possibility to produce sterile immunity [18]. Due to advances in molecular biology and an in-depth knowledge of ASFV, much progress has been made in live attenuated vaccines generated by targeted gene deletion [19]. Numerous gene-deleted ASFVs have shown good efficacy and improved safety profile [20–22], and accompanying genetic tests to discriminate between infected and vaccinated animals were established [23]. Although live attenuated vaccines against ASF displayed good potential, some of them still suffered from severe adverse side effects and safety issues [4, 24]. In contrast, the use of ASFV subunits as vaccines would represent a safer option. Several ASFV proteins, including p54, p30, p72 and CD2v, have been reported to induce neutralizing antibodies in pigs [25]. Recent studies have also shown that binding and internalization of ASFV were inhibited by antibodies to ASFV proteins p30 and p54, respectively [26]. Immunization with a combination of recombinant p30 and p54, or a chimeric protein p54/30 induced effective antibody responses in swine and achieved different degrees of protection against ASFV challenge [27, 28]. Despite these encouraging results, little attention has been paid to enhancing the protective immunity induced by ASFV proteins p30 and p54. As an essential component of protective immunity, cellular immune response also plays a key role in the clearance of ASFV infection [29, 30]. More recently, pigs immunized with an ASFV DNA expression library were shown to be partially protected against a lethal challenge with ASFV as a result of antigen-specific CD8^+^ T cells [30].

The major outer membrane lipoprotein I (OprI) of *Pseudomonas aeruginosa* is a ligand of the Toll-like receptor (TLR)-2. OprI has intrinsic adjuvant properties even fused with other peptides or proteins [31] and potent humoral and cytotoxic T cell responses were induced after immunization with antigens fused to it [32, 33]. The immune responses induced by OprI and OprI-fusions have been attributed to activation of TLR-2 signaling [34]. The immunomodulatory properties of OprI-fusions have also been exploited in the development of vaccines [35] and, recently, immunization with recombinant antigens fused to OprI was shown to significantly inhibit vertical transmission of *Neospora caninum* and postnatal mortality in mice [36].

In the present study, we investigated whether OprI fusion proteins can enhance antigen-specific cellular and humoral immune responses induced by ASFV proteins p30 and p54. We designed two recombinant OprI fusion proteins, as well as three different recombinant proteins based on the sequences of p30 and p54. The immunostimulatory activity of these recombinant proteins was first assessed using murine bone marrow-derived dendritic cells (BMDCs). The humoral and cellular immune responses induced by these potential new vaccine candidates were then evaluated in mice to provide preliminary evidence before proceeding to experiments in swine. To the best of our knowledge, this is the first report of recombinant proteins consisting of ASFV proteins p30 and p54 fused with OprI. We believe that our work will contribute to the development of a novel ASFV subunit vaccine candidate.

## Materials and methods

### Cell culture and virus

Primary porcine alveolar macrophages (PAMs) were obtained from Large White pigs (20–40 kg) that were shown to be negative for porcine respiratory and reproductive syndrome virus, classical swine fever virus, ASFV and pseudorabies virus, as previously described [37]. The PAMs were cultured in RPMI-1640 medium (Thermo Fisher Scientific, MA, USA), supplemented with 15% fetal bovine serum (FBS, Thermo Fisher Scientific), penicillin (100 units/mL) and streptomycin (100 units/mL) (Thermo Fisher Scientific) at 37 °C under an atmosphere containing 5% CO_2_.

ASFV China/Sichuan/2019 (CN/SC/19) was obtained from the Regional Laboratory of African swine fever, Lanzhou Veterinary Research Institute (LVRI, Chinese Academy of Agricultural Sciences, China). The CN/SC/19 stock used for the neutralization tests was propagated and titrated in PAMs.

### Construction of recombinant fusion proteins

The amino acid sequence of *P. aeruginosa* OprI was retrieved from the GenBank database (GenBank X13748.1). The amino acid sequences of p30 and p54 proteins from ASFV-SY18 were also retrieved from GenBank (GenBank MH766894.1). The transmembrane region (His30–Phe52) of p54 was replaced by a long flexible linker “(GGGGS)_3_” to generate modified p54, with p30 remaining unchanged when expressed alone. For the fusion forms, modified p54 was fused to the C-terminus of p30 to construct p30-modified p54 (PM). The PM was then fused to OprI at C-terminus, with or without a universal tetanus toxoid CD4+ T cell epitope P2 (TT-P2) at N-terminus to provide, OprI-p30-modified p54 (OPM) and OprI-p30-modified p54-TT-P2 (OPMT), respectively. To facilitate correct folding of the fusion proteins, the long flexible linker “(GGGGS)_3_” was used to ligate one protein to another, whereas a flexible short peptide “PG” was only used for ligation of TT-P2. All nucleotide sequences corresponding to the constructed proteins were chemically synthesized (GenScript, NanJing, China) as DNA concatemers and cloned into the pET30a(+) expression vector (Novagen, San Diego, CA, USA), which harbors a hexa-histidine tag, through double enzyme digestion with *NdeI* and *XhoI* (New England Biolabs, Ipswich, MA, USA).

### Expression and purification of recombinant fusion proteins

Each expression plasmid was transformed into competent *Escherichia coli* BL21 (DE3) pLysS (Takara, Dalian, China). After identification of positive monoclonal colonies, the resulting expression strains were grown to an optic density (OD) of 0.6–0.8 at 600 nm in Luria broth medium and then induced with isopropylthio-β-galactoside (1 mM) for 4 h at 37 °C. The bacterial pellets were collected by centrifugation and used for protein purification. Two different procedures were used to purify the recombinant proteins. OPM and OPMT were isolated from the bacterial outer membranes as described previously with slight modifications [31]. In brief, bacterial pellets were resuspended in lysis buffer (20 mM Tris–HCl, 50 mM glucose, 10 mM EDTA, pH 8.0) containing lysozyme (2.5 mg/mL) and incubated for 35 min on ice. Following the addition of an equal volume of sarkosyl (2%, w/v), the mixture was ultra-sonicated. The cell lysate was ultra-centrifuged at 100,000 × g for 2 h at 4 °C to obtain insoluble outer membrane pellets. After being treated with chloroform/methanol (2:1, v/v) to extract redundant lipids, the insoluble outer membrane pellets were resuspended in binding buffer (20 mM NaH_2_PO_4_, 300 mM NaCl, 20 mM imidazole, 6 M guanidine hydrochloride, 2% (v/v) triton X-100, pH 8.0) with mild agitation overnight at 4 °C. The supernatants were collected by centrifugation and loaded onto Ni Sepharose resin (GE Healthcare, Chicago, IL, USA). Finally, the bound protein was eluted with elution buffer (20 mM NaH_2_PO_4_, 300 mM NaCl, 8 M urea, 500 mM imidazole, pH 7.5) and refolded by dialysis from 6 M, 4 M, 2 M to 0 M urea in buffer (20 mM NaH_2_PO_4_, 300 mM NaCl, 2 mM β-mercaptoethanol, 0.4% arginine, 10% glycerol, pH 7.5). In contrast, p30, modified p54 and PM were purified from the inclusion bodies of the bacterial pellets, using the same chromatographic process. The concentration of each recombinant protein was measured with the Bradford method. Endotoxin was then removed from the purified proteins using a ToxinEraser™ Endotoxin Removal kit (Genscript, NanJing, China). The endotoxin level in the purified protein samples was determined with a ToxinSensor™ Chromogenic LAL Endotoxin Assay Kit (Genscript, NanJing, China).

### SDS-PAGE and western blotting

Approximately equal amounts of each recombinant protein and bovine serum albumin (as a negative control in western blotting) were mixed with 5 × loading buffer, boiled for 10 min, and then loaded onto 12% SDS-PAGE. The gel was stained with Coomassie brilliant blue R250 (MP Biomedicals, Santa Ana, USA) or electrophoretically transferred to polyvinylidene difluoride membranes (Milipore, San Diego, CA, USA). The membranes were blocked with 5% skim milk for 2 h at room temperature and then incubated with anti-histidine monoclonal antibody (1:5000 dilution, Abcam, Cambridge, UK) or anti-ASFV swine serum (1:300 dilution, Diagnostic Products Center, LVRI, China) overnight at 4 °C. After washing five times with PBS containing 0.05% Tween-20 (PBST), the membranes were incubated with horseradish peroxidase (HRP)-conjugated goat anti-mouse antibody (1:5000 dilution, Abcam) or goat anti-pig antibody (1:5000 dilution, Abcam) for 1 h at room temperature. After being washed with PBST, the membranes were developed using enhanced chemiluminescence reagent (Thermo Fisher Scientific).

### Generation and stimulation of mouse dendritic cells

Specific-pathogen-free (SPF) C57BL/6 mice (6–8 weeks old) were purchased from the Laboratory Animal Center of LVRI. All animal protocols were approved by the Institutional Animal Care and Use Committee of LVRI. BMDCs were generated as described recently [38]. The BMDCs were treated with p30 + modified p54 (10 µg/mL, containing equal amounts of each protein), PM (10 µg/mL), OPM (10 µg/mL), OPMT (10 µg/mL), LPS (0.1 µg/mL) or an equal volume of medium and incubated for 24 h at 37 °C under 5% CO_2_. After centrifugation, tumor necrosis factor-α (TNF-α) and interleukin-12p70 (IL-12p70) were measured in the culture supernatants using commercial ELISA kits (Neobioscience, ShenZhen, China). Cell pellets from each treatment group were washed with PBS, and stained with two groups of fluorescent antibodies (all from BD-Pharmingen, San Diego, CA, USA) for 30 min at 4 °C. One group of antibodies contained phycoerythrin (PE)-conjugated anti-CD11c (clone HL3), allophycocyanin (APC)-conjugated anti-CD40 (clone 3/23) and fluorescein isothiocyanate (FITC)-conjugated anti-CD86 (clone GL1) monoclonal antibodies and the other group contained PE-conjugated anti-CD11c, APC-conjugated anti-CD80 (clone 16-10A1) and FITC-conjugated anti-I-Ab (MHC-II, clone 2G9) monoclonal antibodies. In parallel, BMDCs stained with PE-conjugated anti-CD11c alone were utilized to establish gate boundaries, while unstained BMDCs were used as blank controls. The cells were then washed and resuspended in 100 μL of PBS containing 2% FBS for analysis by flow cytometry using a CytoFlex flow cytometer (Beckman Coulter, Brea, CA, USA). The cells were gated to select the destination cell population. Then, the cells stained with PE-conjugated anti-CD11c alone were used to gate on CD11c-positive cell population within the destination cell population, and the gate limits in the FITC and APC channel were determined in the CD11c-positive cell population. The expression of CD40, CD80, CD86 and MHC-II was analyzed for each sample.

### Immunization of mice

A total of 25 eight-week-old, SPF female BALB/c mice were randomized to five experimental groups (five mice per group). In order to test the immunogenicity of the recombinant proteins in the absence of adjuvants, the mouse in each group was immunized twice with one of the purified recombinant proteins dissolved in PBS buffer (200 μL) by subcutaneous route at 2-week intervals. Mice in groups 1, 2, 3 and 4 received p30 + modified p54 (30 µg, containing equal amounts of each protein), PM (30 µg), OPM (30 µg) and OPMT (30 µg), respectively, and mice in group 5 (control group) received an equal volume of PBS. All mice were bled from the caudal vein at 0, 7, 21 and 28 days post-vaccination (dpv).

### Detection of antibodies to p30 and p54

Antibody responses to p30 and p54 were measured by indirect ELISA. Briefly, 96-microwell plates (Costar, Cambridge, MA, USA) were pre-coated with recombinant p30 (0.125 µg/mL) or modified p54 (0.5 ug/mL) at 100 µL/well in coating buffer (0.1 M carbonate buffer, pH 9.0). After incubation overnight at 4 °C, each plate was blocked with 5% skim milk (200 μL/well) for 2 h at 37 °C and washed five times with PBST. Then, serum samples (1:100 dilutions) from immunized mice were added at 100 μL/well and incubated for 1 h at 37 °C. Positive, negative and blank controls were also established. Subsequent steps were performed in accordance with a conventional protocol. The OD at 450 nm (OD_450_) of each well was detected using a microplate reader (Thermo Fisher Scientific). IgG1 and IgG2a were measured in the same way, except that serum samples were diluted in a twofold series and the secondary antibodies were HRP-labeled goat anti-mouse IgG1 or IgG2a (Abcam) instead of HRP-labeled goat anti-mouse IgG. IgG levels to p30 and p54 were expressed as OD_450_ values. To calculate titers of IgG1 and IgG2a, a cutoff value was defined as the mean specific OD_450_ value plus three standard deviations from mouse sera at 0 dpv, with a dilution of 1:100. The p30- and p54-specific IgG1 and IgG2a titers corresponded to the reciprocal values of the highest dilutions that showed positive.

### Assessment of cytokine expression levels

Three immunized mice from each group were euthanized at 28 dpv and the spleens were removed aseptically and processed to single-cell suspensions. After centrifugation, the cells were treated with erythrocyte lysis buffer and washed twice with PBS. Splenocytes were seeded into 12-well plates (1 × 10^6^ cells/well, 1 mL) and incubated with p30 + modified p54 (10 µg/mL, containing equal amounts of each protein) for 72 h. Culture supernatant was collected from each well and levels of IL-2, interferon-γ (IFN-γ) and TNF-α were detected by commercial ELISA kits (Neobioscience).

### Assessment of lymphocyte proliferation

Splenocytes were obtained as described above and adjusted to 5 × 10^6^ cells/mL in RPMI-1640 medium supplemented with 10% FBS (RPMI-1640 complete medium). Cell suspension (100 μL/well) was inoculated into 96-well plates (Corning) and treated with p30 + modified p54 (5 µg/mL or 10 µg/mL) for 72 h. Wells treated with concanavalin A (ConA, 5 μg/mL, Solarbio, Beijing, China), unstimulated wells and equal amount of RPMI-1640 complete medium were established as positive, negative and blank controls, respectively. After addition of 10 µL of Cell Counting Kit-8 (CCK8) assay solution (APE × BIO, Houston, TX, USA) and incubation for 3.5 h, the OD_450_ of each well was then measured. The results are presented as stimulation index (SI, ratio of the OD_450_ of stimulated well to the OD_450_ of unstimulated well).

### Determination of IFN-γ production by intracellular cytokine staining

Splenocytes (1 × 10^6^ cells/well) were inoculated into 12-well plates (1 × 10^6^ cells/well, 1 mL), followed by stimulation with p30 + modified p54 (10 μg/mL) for 40 h at 37 °C under 5% CO_2_, and then incubated with monensin (1.7 μg/mL, APE × BIO) for another 8 h. The cells were washed three times with PBS and then stained with APC-conjugated anti-CD3 (clone 145-2C11) and FITC-conjugated anti-CD8α (clone 53–6.7) monoclonal antibodies (both from BD Biosciences, San Diego, USA) for 30 min. After being fixed with Fix and Perm reagents (BD Biosciences), the cells were stained with PE-conjugated anti-IFN-γ monoclonal antibody (clone B27, BD Biosciences) for 30 min. The cells were subsequently washed three times with PBS and resuspended in 100 μL of PBS containing 2% FBS for analysis by flow cytometry. CD3-positive cells were gated and the percentage of IFN-γ^+^ CD8^+^ T cells was analyzed for each sample.

### Neutralization assay

The neutralizing ability of sera from mice collected at 0 (pre-immune sera) and 28 dpv (immune sera) was measured as described previously [39]. Sera from each group were incubated for 30 min at 56 °C for complement inactivation. CN/SC/19 (100 PFU) was then incubated overnight at 37 °C with the heat-inactivated sera (dilution: 1/5) or an equal volume of medium (negative control) and inoculated onto PAMs in 24-well plates (150 μL/well). After incubation for 1 h at 37 °C, 0.8% agarose (Lonza Group Ltd., Basel, Switzerland) in Dulbecco's Modified Eagle Medium containing 5% FBS was added to each well (1.5 mL/well) and the plates were further incubated for 5 days. The plates were then stained with 2% crystal violet (prepared in 5% formaldehyde, 0.5 mL/well), and the number of plaques formed on macrophages was compared with the number formed when the virus was mixed with heat-inactivated pre-immune sera. Neutralization percentages were calculated using the following formula:

$$\begin{aligned} & {\text{Neutralization }}\left( \% \right) = {1}00{-}{1}00 \\ & \quad \times {\text{number of plaques in presence of immune serum}} \\ & \quad /{\text{number of plaques in presence of pre}} - {\text{immune serum}}. \\ \end{aligned}$$

### Statistical analyses

All experiments were repeated three times and produced consistent results. Data in the figures are expressed as mean ± standard error of the mean (SEM). The statistical significance of differences between groups was determined by one-way analysis of variance and Student’s t-tests (GraphPad Prism software, San Diego, CA, USA). No significance (ns) between groups was established at *P* > 0.05. *P < 0.05, **P < 0.01 and ***P < 0.001 were considered to be statistically significant differences between groups.

## Results

### Expression and purification of recombinant proteins

**Fig. 1:**
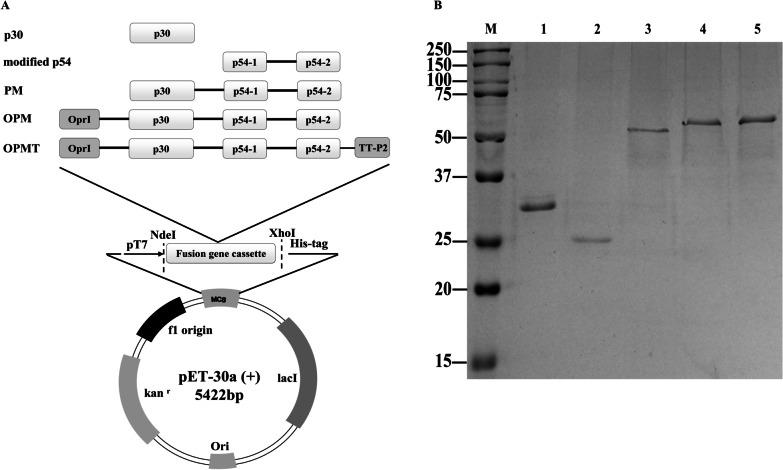
Construction and expression of recombinant fusion proteins. **A** All five constructs, denoted as p30, modified p54, PM, OPM and OPMT, were inserted into expression plasmid pET30a (+) and expressed in *E. coli*. **B** SDS-PAGE analysis of purified recombinant proteins. Lane M, molecular weight markers; lane 1, p30; lane 2, modified p54; lane 3, PM; lane 4, OPM; lane 5, OPMT. Proteins bands were visualized by Coomassie staining

**Fig. 2:**
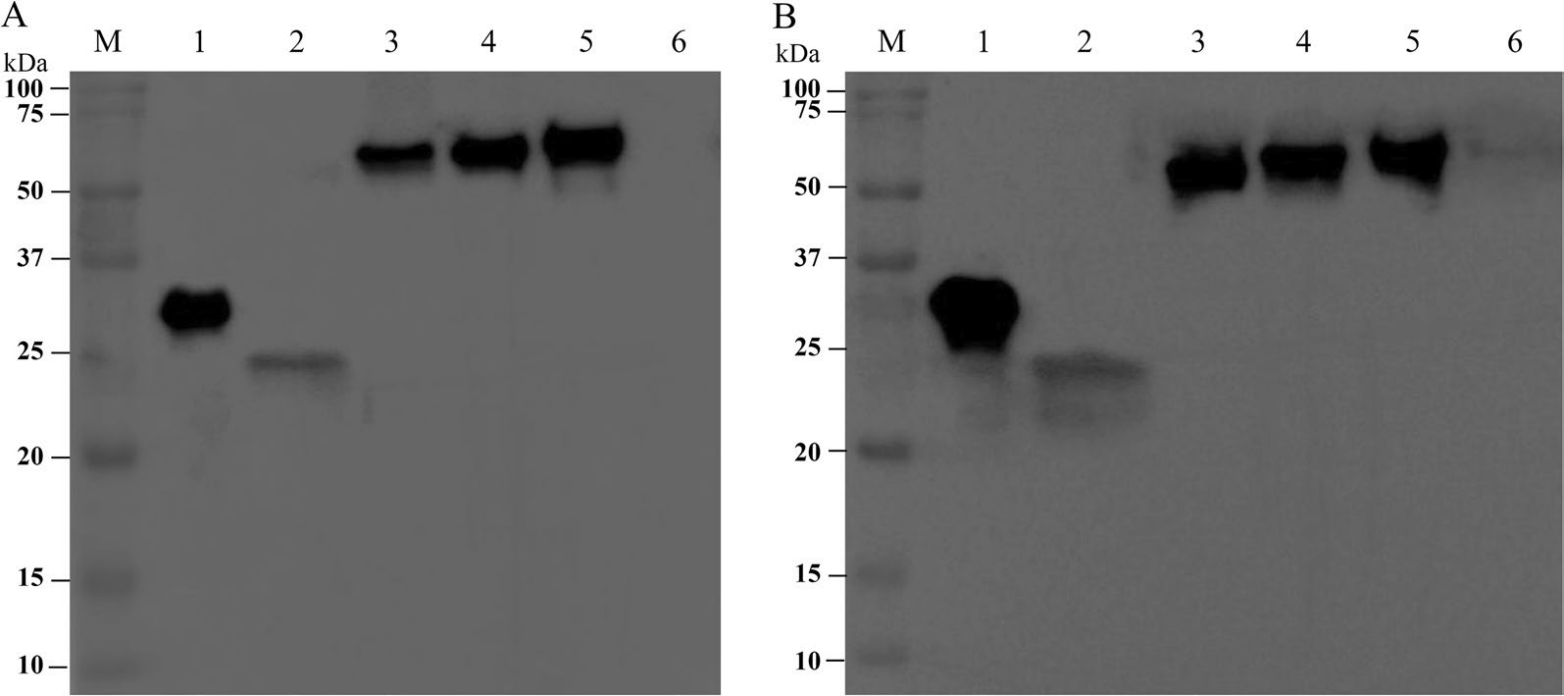
Immunoblot analysis of the recombinant proteins. **A** The recombinant proteins were subjected to western-blotting with 1:5000 diluted anti-polyhistidine monoclonal antibody as primary antibody and 1:5000 diluted goat anti-mouse peroxidase conjugated secondary antibody. **B** Western blotting confirmation of recombinant proteins using 1:300 diluted anti-ASFV swine serum as primary antibody and 1:5000 diluted goat anti-pig peroxidase conjugated secondary antibody. Lane M: molecular weight markers; lane 1, p30; lane 2, modified p54; lane 3, PM; lane 4, OPM; lane 5, OPMT; lane 6, bovine serum albumin

Constructs containing the genes encoding the recombinant proteins were designed and then expressed in *E. coli* (Fig. 1A). These His-tagged recombinant proteins were purified by metal affinity chromatography and confirmed by SDS-PAGE (Fig. 1B). Bands corresponding to p30, modified p54, PM, OPM, OPMT, with molecular weights approximately 26, 30, 56, 63 and 64 kDa, respectively, were clearly visible after staining with Coomassie blue. The recombinant proteins were confirmed by western blotting, using an anti-histidine monoclonal antibody (Fig. 2A) and anti-ASFV swine serum (Fig. 2B). Residual endotoxin in the purified recombinant proteins was detected by Limulus amebocyte lysate assays and shown to be below 0.1 EU/µg.

### Increased stimulation of dendritic cells by OprI-fusion proteins

**Fig. 3:**
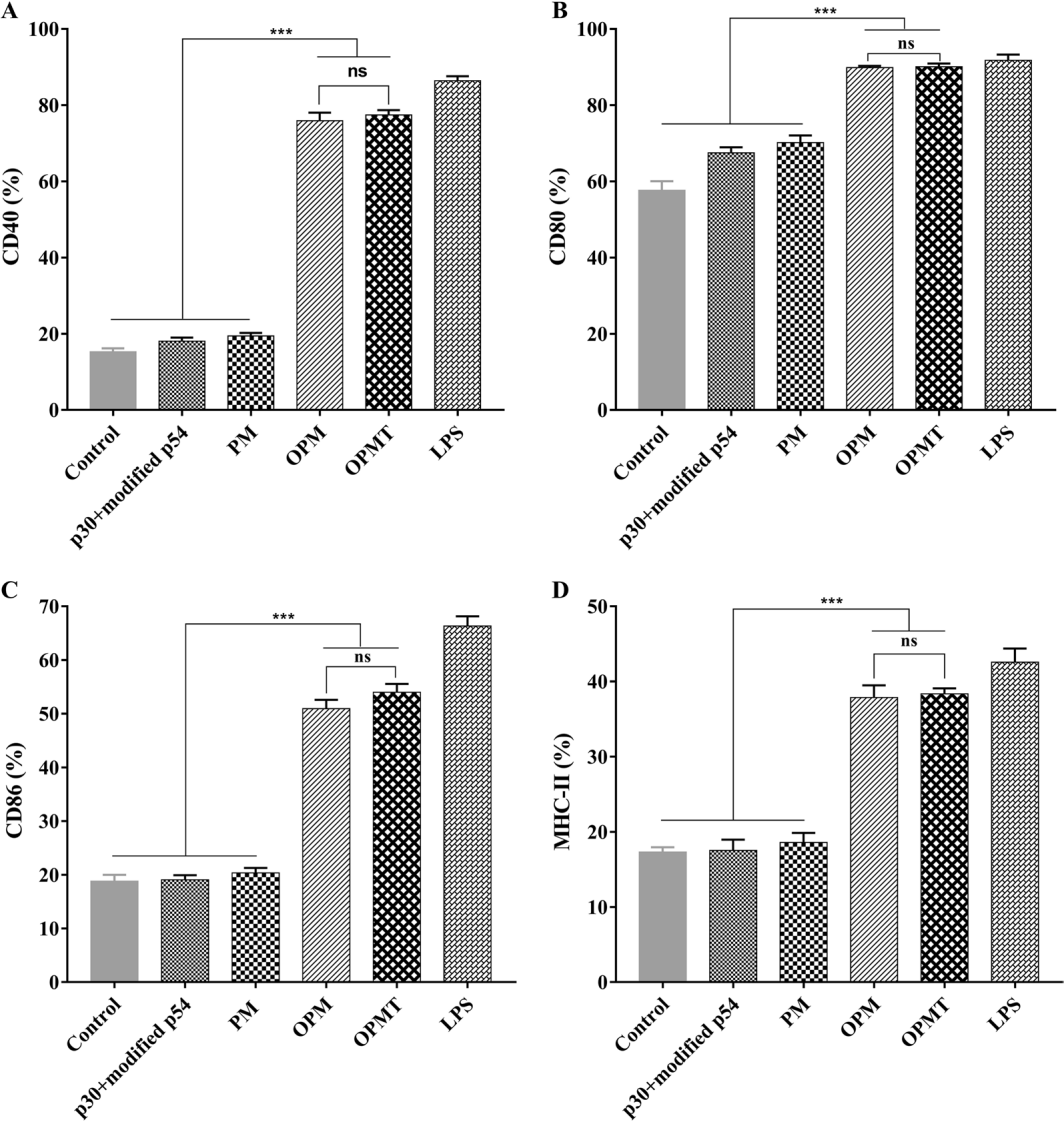
Expression of costimulatory and antigen-presenting molecules by DCs induced by recombinant proteins. BMDCs (10^6^/well) were incubated with p30 + modified p54, PM, OPM or OPMT (10 μg/mL each) for 24 h at 37 °C under 5% CO_2_. RPMI-1640 complete medium and LPS (0.1 µg/mL) served as negative and positive controls, respectively. After staining with anti-CD11c, anti-CD40, anti-CD80, anti-CD86 or anti-MHC-II fluorescent antibodies, the cells were gated on the CD11c-positive population to analyze expression of CD40, CD80, CD86 and MHC-II by flow cytometry. The percentages of CD40 (**A**), CD80 (**B**), CD86 (**C**) and MHC-II (**D**) positive cells in each group were calculated. The graph bars represent mean ± SD (n = 3); ns = *P* > 0.05; *** *P* < 0.001

**Fig. 4:**
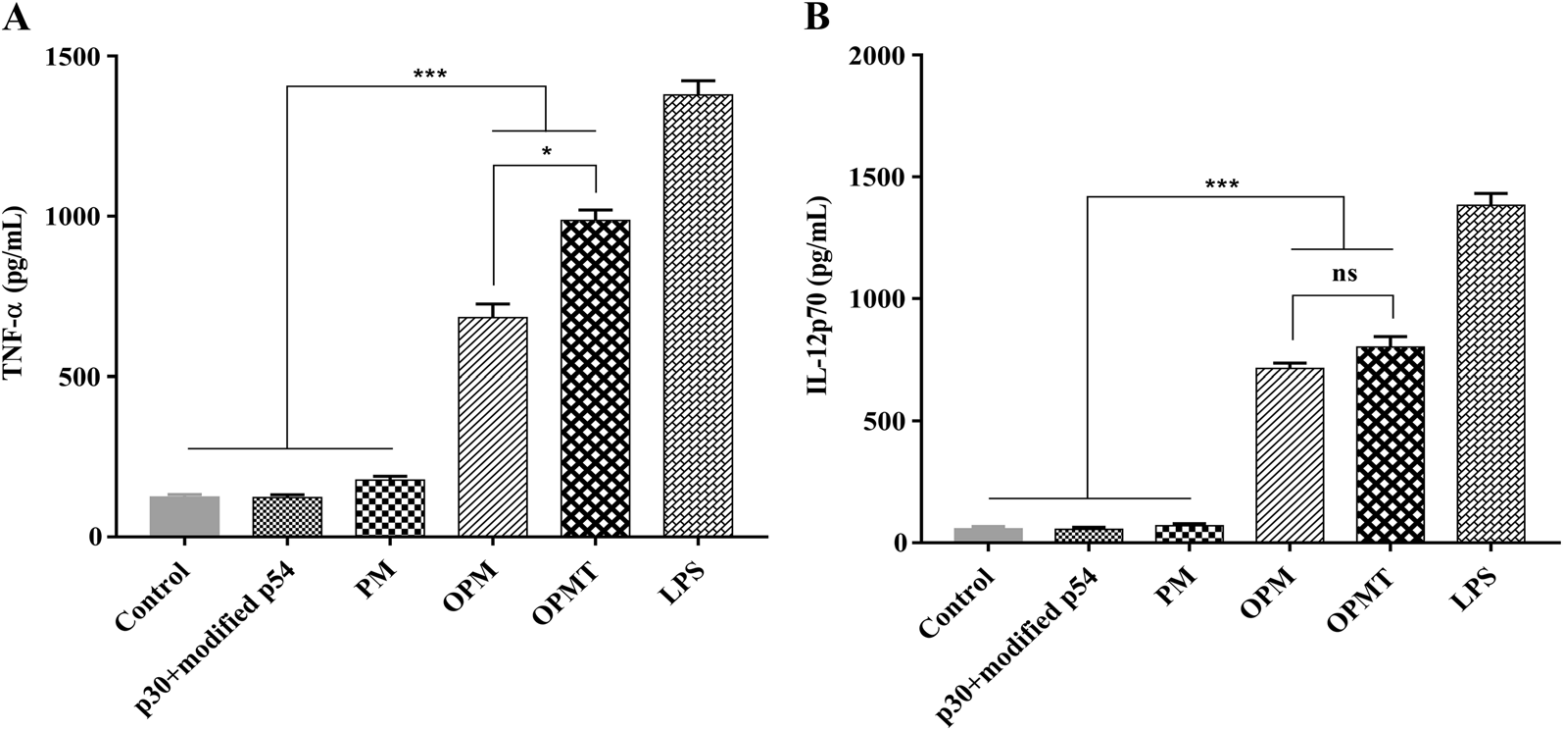
Cytokine production in BMDCs induced by recombinant proteins. BMDCs (10^6^ cells/well) were stimulated with p30 + modified p54, PM, OPM, OPMT (10 μg/mL each) or LPS (0.1 µg/mL) for 24 h at 37 °C under 5% CO_2_. The quantities of TNF-α (**A**) and IL-12p70 (**B**) in the culture supernatant were determined by commercial ELISA kits. All data are displayed as mean ± SD (n = 3); ns = *P* > 0.05; *P < 0.05; ***P < 0.001

The immunostimulatory activity of the purified recombinant proteins was evaluated by determining their effects on dendritic cells (DCs). BMDCs were incubated with each recombinant protein (10 µg/mL), LPS (0.1 µg/mL) or RPMI-1640 complete medium for 24 h. When compared with p30 + modified p54 and PM, both OPM and OPMT induced significant expression of markers critical for DC maturation, including CD40, CD80, CD86 and MHC class II (MHC-II) (all *P* < 0.001; Fig. 3). No significant difference was observed between OPM and OPMT (all *P* > 0.05). The stimulatory effects of OPM and OPMT on DCs were further investigated by measuring the production of proinflammatory cytokines in culture supernatant using commercial ELISA kits. Both OPM and OPMT significantly enhanced secretion of TNF-α and IL-12p70 (both *P* < 0.001), compared with the control groups (Fig. 4).

### Immunization of mice with recombinant OprI-fusion proteins enhanced IgG responses

**Fig. 5:**
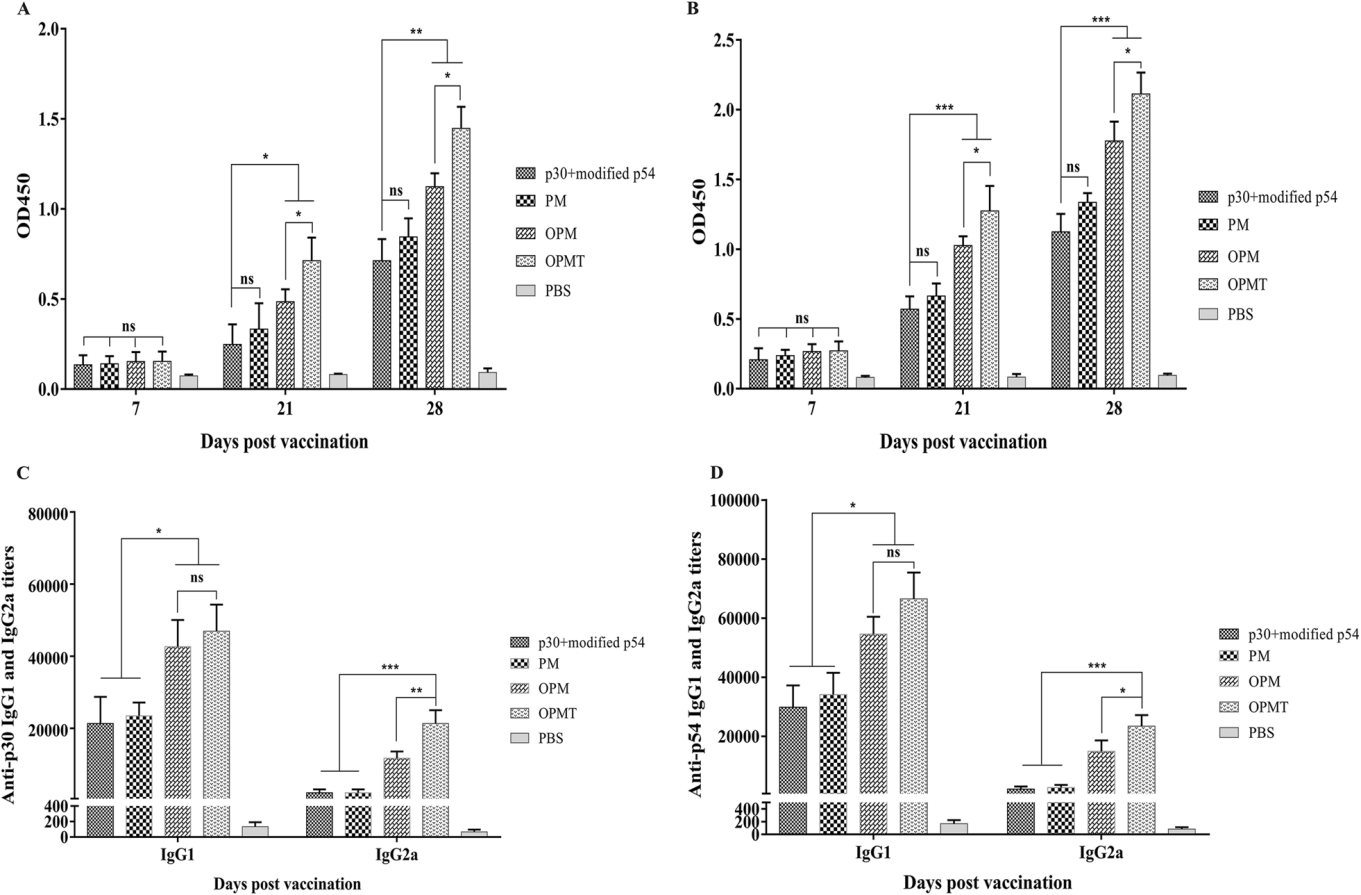
Levels of IgG to p30 and p54 detected by indirect ELISAs. Five BALB/c mice/group were immunized by subcutaneous route with each recombinant protein (30 μg) or an equal volume of PBS. Blood samples were obtained at 0, 7, 21 and 28 dpv. IgG levels to p30 (**A**) and p54 (**B**) of each group at serum dilutions of 1:100 were measured by indirect ELISAs and are expressed as OD_450_ (mean ± SD, n = 5). Characterization of p30-specific (**C**) and p54-specific (**D**) IgG isotype profiles of sera (diluted in twofold series) from immunized mice at 28 dpv by indirect ELISAs. The titer of a given serum sample is defined as the reciprocal value of the highest dilution yielding a positive result and displayed as mean ± SD (n = 3); ns = *P* > 0.05; *P < 0.05; **P < 0.01; ***P < 0.001

IgG responses to p30 and p54 in mice immunized with individual recombinant proteins (30 μg per dose), or an equal volume of PBS, were determined by indirect ELISA. As shown in Fig. 5A, B, p30- and p54-specific IgG responses were detected at 7 dpv in mice that received p30 + modified p54, PM, OPM and OPMT, with no significant difference between them (all *P* > 0.05). After a booster vaccination, however, mice immunized with either OPM or OPMT produced significantly higher levels of IgG against p30 than mice immunized with p30 + modified p54 at 21 (*P* < 0.05) and 28 dpv (*P* < 0.01). Similarly, the p54-specific IgG response in mice immunized with OPM or OPMT was significantly higher than that in mice immunized with p30 + modified p54 at 21 and 28 dpv (*P* < 0.001). There was also no significant difference in levels of p30-specific or p54-specific IgG at the same time point (all *P* > 0.05) between mice that received p30 + modified p54 and those that received PM. IgG responses to p30 and p54 were, however, barely detectable in sera of all mice at 0 dpv (data not shown) and mice that received PBS at any time point. Amongst all the groups, the highest levels of IgG to p30 and p54 at 21 and 28 dpv were generated by mice immunized with OPMT. To analyze the isotypes of anti-p30 and anti-p54 IgG, both IgG1 and IgG2a were determined in the sera of mice at 28 dpv by indirect ELISA, using IgG1- and IgG2a-specific secondary antibodies (Fig. 5C, D). Both the anti-p30 and anti-p54 IgG isotypes in all mice, except those in the control group, were dominated by IgG1. In general, titers of anti-p30 IgG1 in mice that received OPM or OPMT were significantly higher than those in mice that received p30 + modified p54 or PM (*P* < 0.05), with no significant difference between mice immunized with OPM and those immunized with OPMT (*P* > 0.05). The titers of anti-p54 IgG1 in all groups showed a similar trend to titers of anti-p30 IgG1. Additionally, titers of anti-p30 and anti-p54 IgG2a were significantly higher in mice immunized with OPMT than in the other groups. These results suggest that OPMT induces potent anti-p30 and anti-p54 IgG responses in mice and that the responses are dominated by IgG1, but with a certain proportion of IgG2a.

### Assessment of cytokine levels and lymphocyte proliferation elicited by the recombinant proteins

**Fig. 6:**
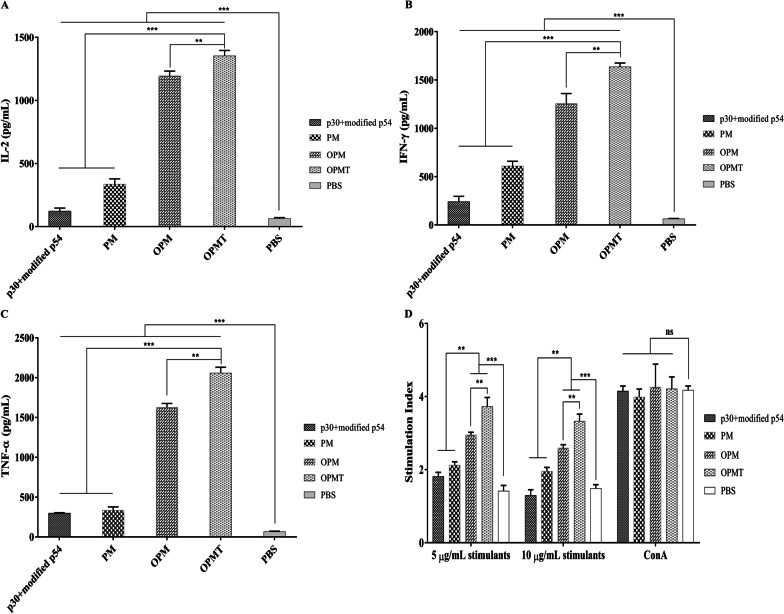
Cytokine levels and T lymphocyte proliferation in splenocytes from mice that received recombinant proteins or PBS. IL-2 (**A**), IFN-γ (**B**) and TNF-α (**C**) produced in splenocytes from mice immunized with each recombinant protein or PBS were detected using commercial ELISA kits. Each value represents the mean ± SD of cytokine production in splenocytes from three individual mice. T lymphocyte proliferation of immunized mice was assessed by CCK8 assays and is presented as a stimulation index (**D**). The graphs show mean results with error bars indicating the SD (n = 3); ns = *P* > 0.05; **P < 0.01; ***P < 0.001

The profiles of IL-2, IFN-γ and TNF-α secretion in culture supernatant of spleen cells from mice in each group were assessed by ELISA. As shown in Fig. 6A, 6B and 6C, levels of all three cytokine in the control group were negligible and significantly lower than those in the other groups (all *P* < 0.001). Splenocytes from mice that received OPMT produced the highest levels of IL-2 (1352 ± 44 pg/mL), IFN-γ (1635 ± 40 pg/mL) and TNF-α (2057 ± 73 pg/mL). Levels of all three cytokine were notably higher in splenocytes from mice immunized with OPM than in splenocytes from mice immunized with p30 + modified p54 and PM. These results indicate that mice immunized with the OprI-fusion proteins produced high levels of IL-2, IFN-γ and TNF-α, which probably contributed to enhancing cellular immunity. To further determine the cellular immune responses induced by the recombinant proteins, T lymphocyte proliferative responses were detected by CCK8 lymphocyte proliferation assays. As shown in Fig. 6D, splenocytes from mice that received either OPM or OPMT showed significantly higher levels of T lymphocyte proliferation after treatment with stimulants (p30 + modified p54 5 μg/mL), compared with splenocytes from mice immunized with p30 + modified p54, PM (both *P* < 0.01) or PBS (*P* < 0.001). Interestingly, T lymphocyte proliferation levels in all groups, except the control group, were slightly lower when treated with a higher concentration of the same stimulants (10 μg/mL), but spleen cells from mice immunized with OPMT still generated a higher level of T lymphocyte proliferation than those from the other groups.

### OprI-fusion proteins increased IFN-γ production from CD8+ T cells

**Fig. 7:**
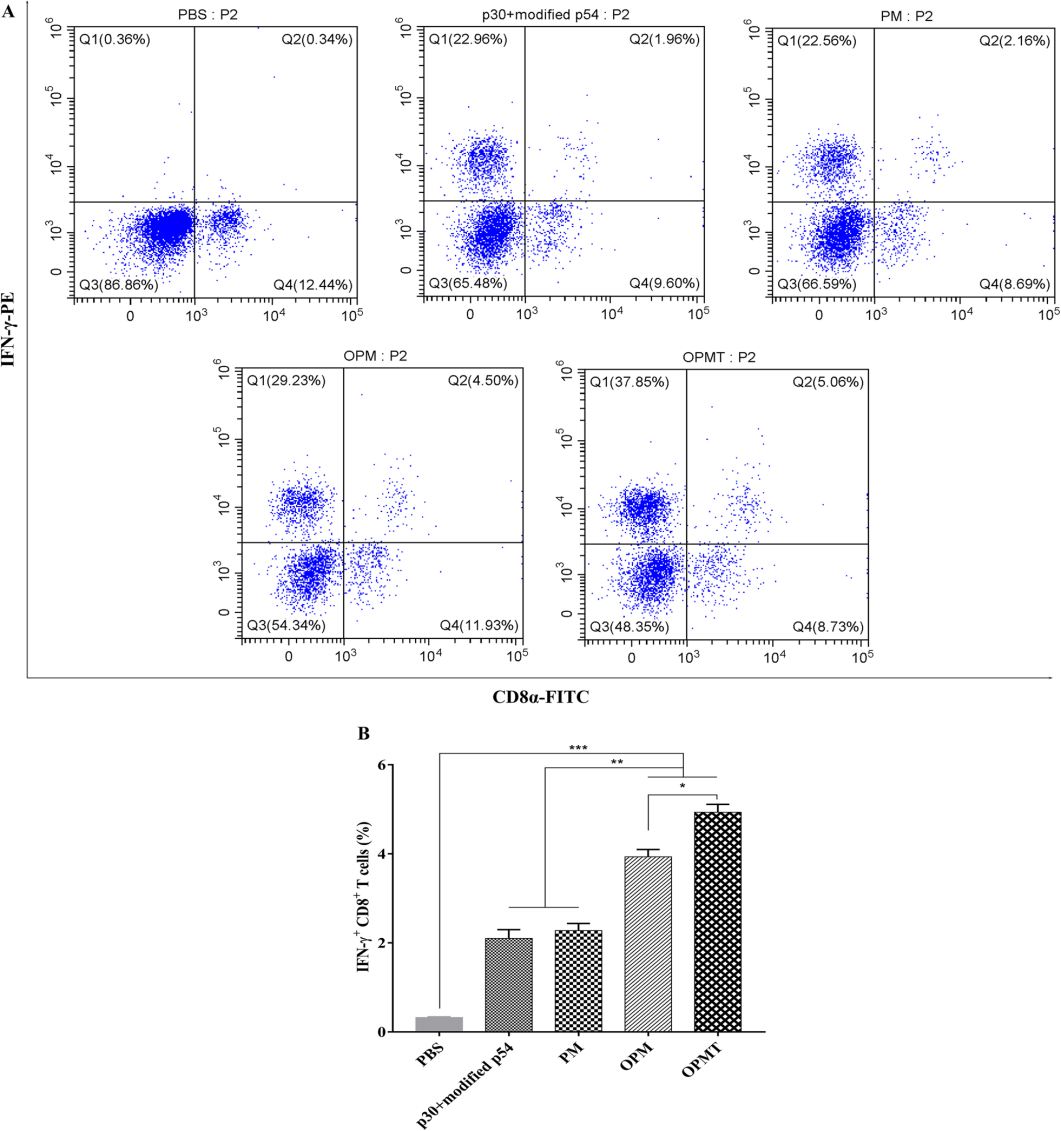
Intracellular cytokine expression in splenocytes from mice that received recombinant proteins or PBS. (**A**) Splenocytes from mice in each group were isolated at 28 dpv and stimulated with p30 + modified p54 (10 μg/mL) for 40 h and incubated with monensin (1.7 μg/mL) for another 8 h at 37 °C under 5% CO_2_. After staining with APC-conjugated anti-CD3, FITC-conjugated anti-CD8α and PE-conjugated anti-IFN-γ antibodies, the cells were gated to select CD3^+^ T lymphocyte (represented by p2) and the percentages of IFN-γ^+^ and CD8^+^ T cells in CD3^+^ T lymphocyte were analyzed by flow cytometry. (**B**) Calculated percentages of IFN-γ^+^ CD8^+^ T cells from three separate experiments. Chart shows means ± SD. *P < 0.05; **P < 0.01; ***P < 0.001

In order to further evaluate cellular immunity in mice immunized with the recombinant proteins, we determined the percentage of IFN-γ^+^ CD8^+^ T cells in spleen cells after re-stimulation by flow cytometry. IFN-γ^+^ CD8^+^ T cells constituted only a small fraction of T cells in any group of the mice (Fig. 7A). The percentages of IFN-γ^+^ CD8^+^ T cells were, however, higher in splenocytes from mice immunized with recombinant proteins, especially OPM and OPMT, than in splenocytes from mice that received PBS. The percentages of IFN-γ^+^ CD8^+^ T cells were determined in three independent experiments (Fig. 7B). Mice immunized with either OPM or OPMT had significantly higher percentages of IFN-γ^+^ CD8^+^ T cells than mice immunized with p30 + modified p54 or PM (*P* < 0.01). It is noteworthy that the percentage of IFN-γ^+^ CD8^+^ T cells in mice that received OPMT was higher than in mice that received OPM. These data demonstrate that immunization of mice with OprI-fusion proteins increased the proportion of IFN-γ^+^ CD8^+^ T cells, indicating activation of T cells in the mouse model.

### Sera from immunized mice neutralize ASFV infection in vitro

**Fig. 8:**
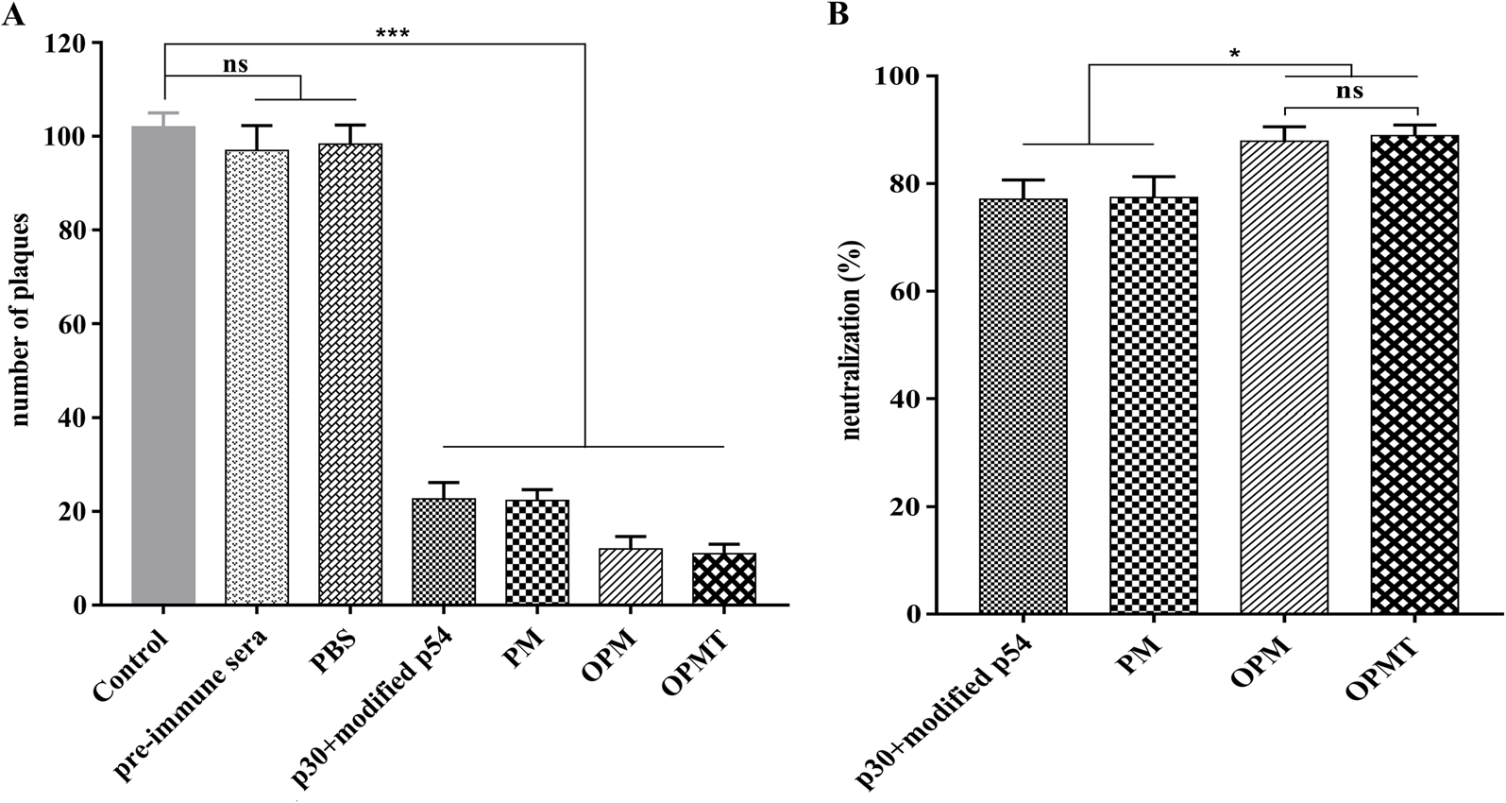
Neutralization of ASFV infection by sera from immunized and non-immunized mice. CN/SC/19 was pre-incubated with heat-inactivated pre-immune sera, immune sera (both at 1:5 dilution) or an equal volume of medium and used to infect PAMs. Viral production was measured at 5 days after infection by plaque assay. **A** Numbers of lysis plaques were counted and presented as means ± SD (n = 3). **B** Neutralization percentages of sera from mice that received each recombinant protein were calculated. Chart shows average of sera from three mice in each group with standard error; ns = *P* > 0.05; *P < 0.05; ***P < 0.001

The presence of neutralizing antibodies against ASFV in the sera from immunized mice was analyzed by measuring plaque-forming units of ASFV after incubation with the heat-inactivated serum from immunized mice at 28 dpv or from non-immunized mice. Compared with the control group, sera from mice immunized with any of the recombinant proteins significantly decreased ASFV plaque formation (*P* < 0.001), indicating that ASFV was neutralized by these immunized sera (Fig. 8A). In contrast, sera from PBS-immunized and non-immunized mice did not reduce ASFV plaque formation. The percentages of virus neutralized by the sera from mice that received the recombinant proteins were calculated based on the results of the plaque assays. The average percentages of virus neutralized by the sera from mice immunized with p30 + modified p54, PM, OPM and OPMT were 77.1%, 77.4%, 86.8% and 87.9%, respectively (Fig. 8B). These results confirm the neutralizing activity of antibodies induced by all of the recombinant proteins used in our study. More importantly, antibodies from mice that received either OPM or OPMT showed enhanced ability to neutralize ASFV infectivity in vitro.

## Discussion

The use of ASFV proteins p30 and p54 as a subunit vaccine has been explored in different works. Earlier studies showed that vaccination with a combination of recombinant p30 and p54 or a chimeric protein p54/30 conferred partial protection against ASFV challenge in swine due to neutralizing antibodies [27]. More recently, another study reported that immunization of pigs with ASFV proteins p30, p54, p72 and p22 induced insufficient protection, with only a 2-day delay to onset of clinical disease after challenge [40]. The controversial results in these studies are probably due to the existence of interfering antibodies or varied experimental setups [41], which suggested the importance of selecting suitable ASFV antigens and of the protective humoral and cellular immunity induced by these antigens. In the present study, we designed two novel fusion constructs, OPM and OPMT, consisting of p30 and modified p54 genetically fused to OprI with or without a universal CD4^+^ T cell epitope. On the one hand, OprI, targeting TLR-2, can thus be efficiently taken up by monocytes/macrophages in vivo and enhance antigen-specific immune responses [32]. In this way, OprI fulfills the role of a molecular adjuvant and has shown to improve vaccine efficacy when fused with antigens or admixed [36, 42]. The application of OprI in fusion proteins has been extended to the antigen encoded by *G1340L* of ASFV and the resulting protein was capable of inducing ASFV-specific cytotoxic T lymphocytes [43]. On the other hand, tetanus toxoid has been extensively studied as a carrier protein of conjugate vaccines against the bacteria *Haemophilus influenzae type b*, *Neisseria meningitidis*, and *Streptococcus pneumoniae* and shown to be universally immunogenic in humans and mice [44]. More recently, the P2 universal CD4^+^ T cell epitope (aa 830–844) of tetanus toxoid (TT-P2), has been demonstrated to enhance the immunogenicity of a peptide vaccine for malaria [45] and an epitope-based vaccine for rotavirus [46] in mice or guinea pigs. Hence, TT-P2 was utilized as an efficient carrier protein to enhance cellular and humoral immune response in vaccine design [47, 48]. The main purpose of our study is to develop a novel ASFV subunit vaccine candidate by combining two of the most antigenic and immunogenic ASFV proteins and then further enhancing protective immune responses by combining it with OprI and TT-P2 in fusion proteins.

We prepared and purified five recombinant proteins. The majority of the p30, modified p54 and PM was found in inclusion bodies and the proteins were successfully purified using conventional procedures. Almost all of the OPM and OPMT were also found in insoluble pellet fractions after sonication, but neither of them could be acquired using the same purification procedures (data not shown). After a major modification of the purification protocol, we finally obtained OPM and OPMT from the insoluble outer membranes, indicating that OPM and OPMT were secreted to the cell membrane, under the guidance of the signal peptide in OprI [31]. In addition, another obvious difference is that only about 50% of OprI-fusion proteins were recovered in endotoxin-removal process with polymyxin B columns. Considering possible value of OprI-fusion proteins in the development of ASF vaccines, it is important to improve its recovery rate. As previously reported, hot phenol/water might be a better choice to extract endotoxin from OprI-fusion proteins prior to metal affinity chromatography [49]. All five recombinant proteins were recognized by anti-ASFV swine serum, suggesting that these recombinant proteins remained immunoreactive, even in different fusion forms. These findings are consistent with the excellent properties of OprI, the coding sequence of which accepts exogenous sequences at the C-terminal without affecting the integrity of the protein [35].

Since DCs are the most potent and versatile antigen-presenting cells in the immune system [50], we used murine BMDCs to evaluate the stimulatory activity of the recombinant proteins. Both OPM and OPMT were able to activate DCs, as demonstrated by upregulation of antigen-presenting molecules (MHC-II) and costimulatory molecules (CD40, CD80 and CD86), which is in accordance with the effect of OprI alone on DCs [42]. DCs activated by OPM and OPMT secreted higher levels of both TNF-α and IL-12p70, driving T-cell polarization towards the Th1 or Th2 lineage. The level of TNF-α induced by OPM and OPMT is similar to OprI and OprI-fusion proteins in two separate reports, but lower than those of another study [36, 51, 52]. The most likely reason for this is that the incubation time in the latter study was longer. Interestingly, OPM and OPMT induced a much higher level of IL-12p70 than those of the other studies above. This may be due to a synergistic interaction between the sequences of p30 or p54 and OprI. We also found that the maturation and activation of DCs triggered by OPM and OPMT were due to the presence of OprI. The role of OprI in the activation of DCs has been attributed to activation of the TLR2/4 signaling pathway [51]. These results suggest that both OPM and OPMT can activate DCs in a similar way to OprI, and may thus contribute to the activation of T-cell mediated immune responses.

Because humoral immune responses are important to defend against ASFV infection [53, 54], we examined the humoral immune responses in mice elicited by the recombinant proteins. We found that OPM and OPMT were highly immunogenic and that vaccinations with these proteins induced significantly higher levels of IgG to p30 and p54 than any other vaccinations, in the absence of adjuvants. In line with our findings, previous studies showed that the production of specific antibodies was strongly enhanced by the fusion of OprI [52]. The levels of IgG to p30 and p54 induced by OPMT were higher than those induced by OPM under the same conditions, suggesting that TT-P2 in combination with OprI leads to a synergistic enhancement in induction of humoral response. Analysis of IgG isotypes showed that both anti-p30 and anti-p54 IgG isotypes in the sera of mice vaccinated with all of the recombinant proteins were dominated by IgG1. This is consistent with some studies [35, 36], while others reported that OprI-fusion proteins induced a more balanced IgG1 and IgG2 responses in mice [33, 52]. The most likely reason for this discrepancy is the varied sequences fused to OprI. The antibody titers of IgG1 and IgG2a elicited by OPM and OPMT were significantly higher than the titers induced by the other recombinant proteins, suggesting that OprI-fusion proteins induce a mixed Th1/Th2 profile response. We also found that in comparison with OPM, the TT-P2 component in OPMT contributed to enhanced induction of IgG2a. Our results demonstrate that robust humoral immune responses were induced by OprI-fusion proteins and also increase our understanding of the function of OprI and TT-P2 when fused to viral proteins.

Since it has been shown that cellular immune responses make a large contribution to protective immunity against intracellular infections with ASFV [30, 55], we measured IL-2, IFN-γ and TNF-α secreted by spleen cells after re-stimulation. Splenocytes from mice immunized with either OPM or OPMT had the capacity to upregulate secretion of cytokines involved in lymphocyte proliferation (IL-2), Th1 stimulation (IFN-γ) and inflammation (TNF-α). The level of IFN-γ secreted by splenocytes from mice vaccinated with either OPM or OPMT was similar to that of OprI-fusion proteins immunized mice in previous researches [36, 51], favoring Th1 profile response. In other studies, OprI-fusion proteins induced a either lower or higher level of IFN-γ as a result of different experimental designs and sequences fused to OprI [33, 52]. As another important cytokines related to cellular immunity, the level of IL-2 induced by OprI-fusion proteins was also assessed in some previous studies [36, 56], but was slightly lower than that of mice immunized with OPM or OPMT in our study. Our data also showed that OPM and OPMT elicited a high level of TNF-α, which were shown to play a key role in cellular immune responses induced by OprI-based vaccines [33]. These results indicate that OprI-fusion proteins strongly stimulate immune cells in immunized mice. As a consequence of the inclusion of TT-P2, spleen cells from mice immunized with OPMT produced higher levels of IL-2, IFN-γ and TNF-α than spleen cells from mice immunized with OPM. T lymphocyte proliferative responses in the mice were determined to further assess the cellular immunity induced by the recombinant proteins. In accordance with cytokines secreted by splenocytes, T lymphocyte proliferation level in the mice immunized with OPMT was significantly higher than that in mice immunized with the other proteins. IFN-γ^+^ CD8^+^ T cells, which represent a fraction of functional CD8^+^ T cells, played a key role in promoting antigen-specific proliferation of CD8^+^ T cells to clear viral infection [57]. Fascinatingly, we found that the proportion of IFN-γ^+^ CD8^+^ T cells in T lymphocyte from mice immunized with OPMT was notably higher than that in T lymphocyte from any other group, indicating that immunization with OPMT favors activation of T cells. Taken together, these results suggest that immunization with OPMT generated strong, antigen-specific cellular immunity.

Although higher levels of IgG were detected by ELISA in the sera from mice immunized with either OPM or OPMT, it was not known whether the immune sera would be effective in neutralizing the virus. We therefore established an in vitro neutralization assay as previously described [26, 58], to analyze neutralizing capability of the immune sera. The sera from mice immunized with all recombinant proteins were able to neutralize ASFV in vitro. On the one hand, the percentage of ASFV neutralized by the sera from the mice that received p30 + modified p54 were similar to that of previous studies [39], in which recombinant proteins p30 and p54 were expressed in baculovirus. This indicates that despite the difference in protein expression and purification procedures, neutralizing antibodies induced by the recombinant p30 and p54 are not affected. On the other hand, either OPM or OPMT exhibited higher neutralizing capability, indicating that the OprI fusion strategy could enhance the induction of neutralizing antibodies by p30 and p54 due to its adjuvant effect. Interestingly, although OPMT induced higher levels of IgG to p30 and p54 than OPM, the virus was neutralized equally well by sera from mice immunized with either of them. This is likely because IgG1 plays a dominant role in neutralization of ASFV. IgG1 has also been shown to be more effective in neutralizing other viruses [59, 60]. We also found that ASFV was not completely neutralized, even by sera from mice immunized with OPMT. Similar observations in neutralization assays of ASFV were reported previously [61, 62], probably as a result of the complex structure of ASFV particles. In this study, we have demonstrated that immunization of mice with either OPM or OPMT was better able to induce neutralizing antibodies against ASFV, indicating that OprI-adjuvanted subunits could empirically improve subunit-induced protection. In our next study, we will carry out more comprehensive animal experiments with OPM or OPMT to evaluate the effects of vaccination routes, dose and adjuvant on immune responses and to assess the protective efficacy against lethal ASFV challenge in pigs. Considering the limited range and intensity of immune responses induced by a single component, the vaccines containing a cocktail of OprI-fused ASFV proteins combined with a heterologous prime-boost strategy may elicit comprehensive humoral and cellular immunity against ASFV challenge. Several prime-boost vaccination approaches including DNA prime and recombinant vaccinia virus boost, modified Vaccinia Ankara virus prime and proteins boost, and recombinant alphavirus prime and attenuated ASFV boost, have been evaluated for the development of vaccines against ASF [63–65], which will provide references for our future studies.

## Conclusion

We have demonstrated that OprI-fused proteins, generated by genetic fusion, maintained the immunogenic properties of ASFV proteins and the immunostimulatory activity of OprI. Furthermore, immunization of mice with either OPM or OPMT in the absence of adjuvants resulted in potent antigen-specific T and B cell responses in vivo and the antibodies induced by them neutralized more than 86% of ASFV in vitro. In comparison with OPM, OPMT was more effective in eliciting cellular immune responses. Although further exploration on how best to enrich and optimize the components of OPMT as a subunit vaccine is needed, we have shown that fusing OprI, together with a universal T cell epitope, to viral proteins provides a promising approach to develop novel subunit vaccines against ASF in the future.

## Data Availability

The datasets used and/or analyzed in this study are obtained and available from the corresponding authors upon a reasonable request.

## Abbreviations

ASF: African swine fever
ASFV: African swine fever virus
OprI: Major outer membrane lipoprotein I
TLR: Toll-like receptor
BMDCs: Bone marrow-derived dendritic cells
PAMs: Porcine alveolar macrophages
FBS: Fetal bovine serum
LVRI: Lanzhou Veterinary Research Institute
TT-P2: A universal tetanus toxoid CD4+ T cell epitope P2
PM: P30-modified p54
OPM: OprI-p30-modified p54
OPMT: OprI-p30-modified p54-TT-P2
OD: Optic density
EDTA: Ethylenediaminetetraacetic acid
LAL: Limulus amebocyte lysate
SDS-PAGE: Sodium dodecyl sulfate polyacrylamide gel electrophoresis
PBS: Phosphate buffer saline
PBST: PBS containing 0.05% Tween-20
SPF: Specific-pathogen-free
TNF-α: Tumor necrosis factor-α
IL-12p70: Interleukin-12p70
IFN-γ: Interferon-γ
PE: Phycoerythrin
FITC: Fluorescein isothiocyanate
APC: Allophycocyanin
CD: Cluster of differentiation
MHC-II: Major histocompatibility complex class II
dpv: Days post-vaccination
ELISA: Enzyme-linked immunosorbent assay
IgG: Immunoglobulin G
ConA: Concanavalin A
CCK8: Cell Counting Kit-8
SEM: Standard error of the mean
ns: No significance
DCs: Dendritic cells
LPS: Lipopolysaccharide
